# Electrocoagulation process to Chemical and Biological Oxygen Demand treatment from carwash grey water in Ahvaz megacity, Iran

**DOI:** 10.1016/j.dib.2017.03.006

**Published:** 2017-03-09

**Authors:** Mohammad Javad Mohammadi, Afshin Takdastan, Sahand Jorfi, Abdolkazem Neisi, Majid Farhadi, Ahmad Reza Yari, Sina Dobaradaran, Yusef Omidi Khaniabadi

**Affiliations:** a Abadan school of Medical Sciences, Abadan, Iran; b Student Research Committee, Department of Environmental Health Engineering, School of Public Health and Environmental Technologies Research Center, Ahvaz Jundishapur University of Medical Sciences, Ahvaz, Iran; c Environmental Technologies Research Center, Ahvaz Jundishapur University of Medical Sciences, Ahvaz, Iran; d Department of Environmental Health Engineering, School of Public Health, Ahvaz Jundishapur University of Medical Sciences, Ahvaz, Iran; e Environmental health Engineering, school of health, Ahvaz Jundishapur University of Medical Sciences, Ahvaz, Iran; f Research Center for Environmental Pollutants, Qom University of Medical Sciences, Qom, Iran; g Department of Environmental Health Engineering, Faculty of Health, Bushehr University of Medical Sciences, Bushehr, Iran; h The Persian Gulf Marine Biotechnology Research Center, The Persian Gulf Biomedical Sciences Research Institute, Bushehr University of Medical Sciences, Bushehr, Iran; i Health Care System of Karoon, Ahvaz Jundishapur University of Medical Sciences, Ahvaz, Iran

**Keywords:** Grey water effluent, Electrocoagulation, COD removal, BOD removal

## Abstract

In this work, we present the result of an electric coagulation process with iron and aluminum electrodes for removal of chemical and biological oxygen demand (COD and BOD) from grey water in different car washes of Ahvaz, Iran. Nowadays, one of the important dangerous that can contaminate water resources for drinking, agriculture and industrial is Car wash effluent [1,2]. In this study, initial COD and BOD concentration, pH of the solution, voltage power and reaction time was investigated. The concentration level of remaining COD and BOD in samples was measured, using DR/5000 UV–vis HACH spectrophotometer [3,4]. The effects of contact time, initial pH, electrical potential and voltage data on removal of COD and BOD were presented. Statistical analysis of the data was carried out using Special Package for Social Sciences (SPSS 16).

**Specifications Table**

**Table:** 

*Subject area*	*Environment*
*More specific subject area*	*Chemical and biological oxygen demand*
*Type of data*	*Table, figure*
*How data was acquired*	*DR/5000 UV–vis HACH spectrophotometer*
*Data format*	*Raw, analyzed*
*Experimental factors*	– *For samples collection from different grey water of Ahvaz, a glass tank was used with a volume of 2–4 l, containing 3 electrode-plate iron and aluminum (Al-Al, Al-Fe, Fe-Fe)* was *used for* Electrocoagulation *removal.* – *After collection of wastewater along the car washes, added Sulfuric acid (H*_*2*_*SO*_*4*_*), potassium dichromate (K*_*2*_*Cr*_*2*_*O*_*7*_*), mercury sulfate (HgSO*_*4*_*), silver sulfate (Ag*_*2*_*SO*_*4*_*), potassium hydrogen phthalate (C*_*8*_*H*_*5*_*KO*_*4*_*) and 3-methyl-2-benzothiazoline, then it was stored in a dark place at 4 °C temperature until the metals analysis* – *The effects of contact times, initial pH, electrical potential and voltage were examined.*
*Experimental features*	*Electrocoagulation between many treatment processes having to be cost-effective for wastewater treatment with pollutant wide range.*
*Data source location*	*Ahvaz, Iran*
*Data accessibility*	*Data is with this article.*

**Value of the data**

• These data describe changes in COD and BOD removal from grey water by electrocoagulation process.
• Data show that electrocoagulation can be used as cost-effective for removal of other pollutant from wastewater.
• Data of this study can be used to design the electrocoagulation experiments for removal of wide range of pollutant in wastewater.
• Data are important for discharge environment especially resource water, aqueous and agriculture.

## 1 Data

**Fig. 1:**
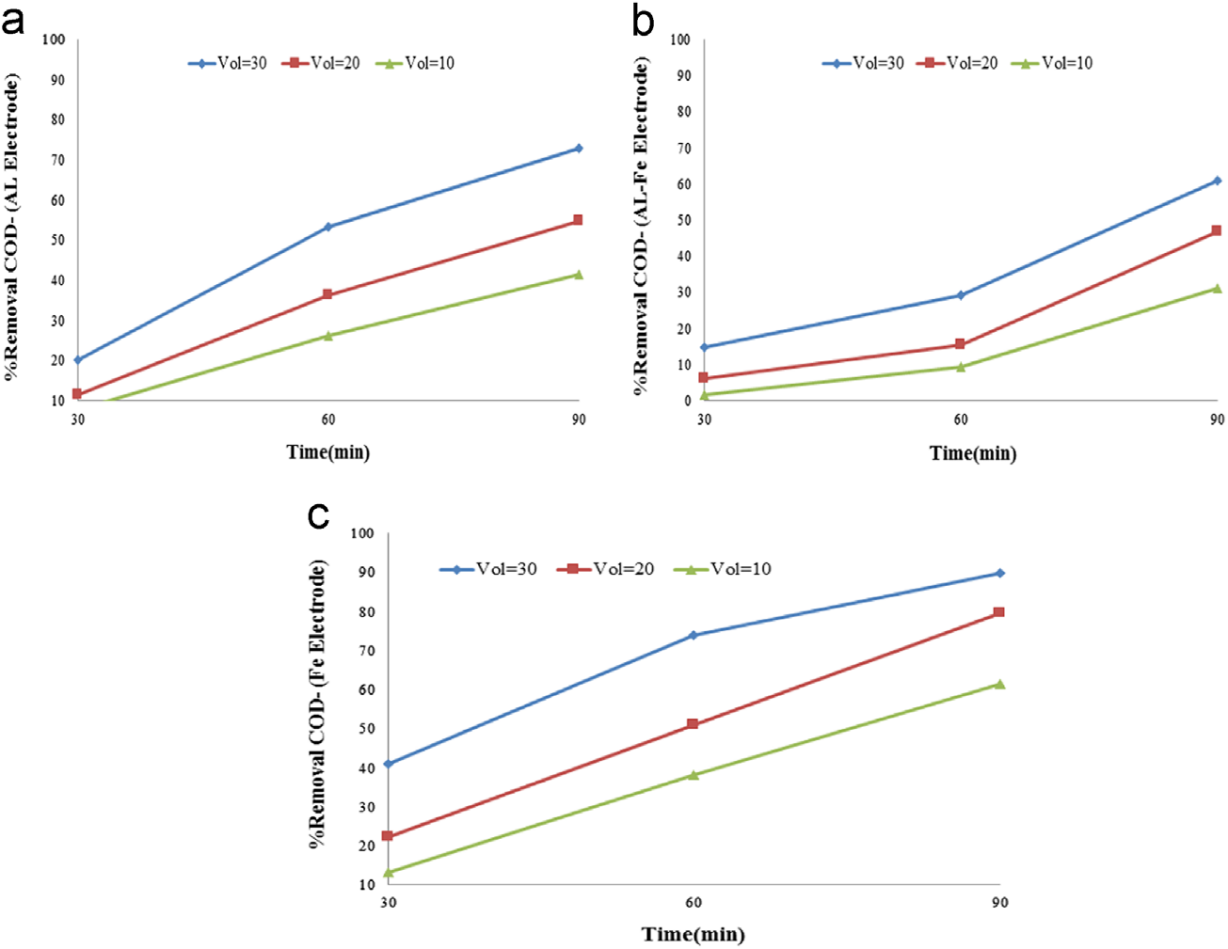
(a) Aluminum electrode, (b) Aluminum – Iron electrode, and (c) Iron electrode applied in the different Voltage on COD removal efficiency .

**Fig. 2:**
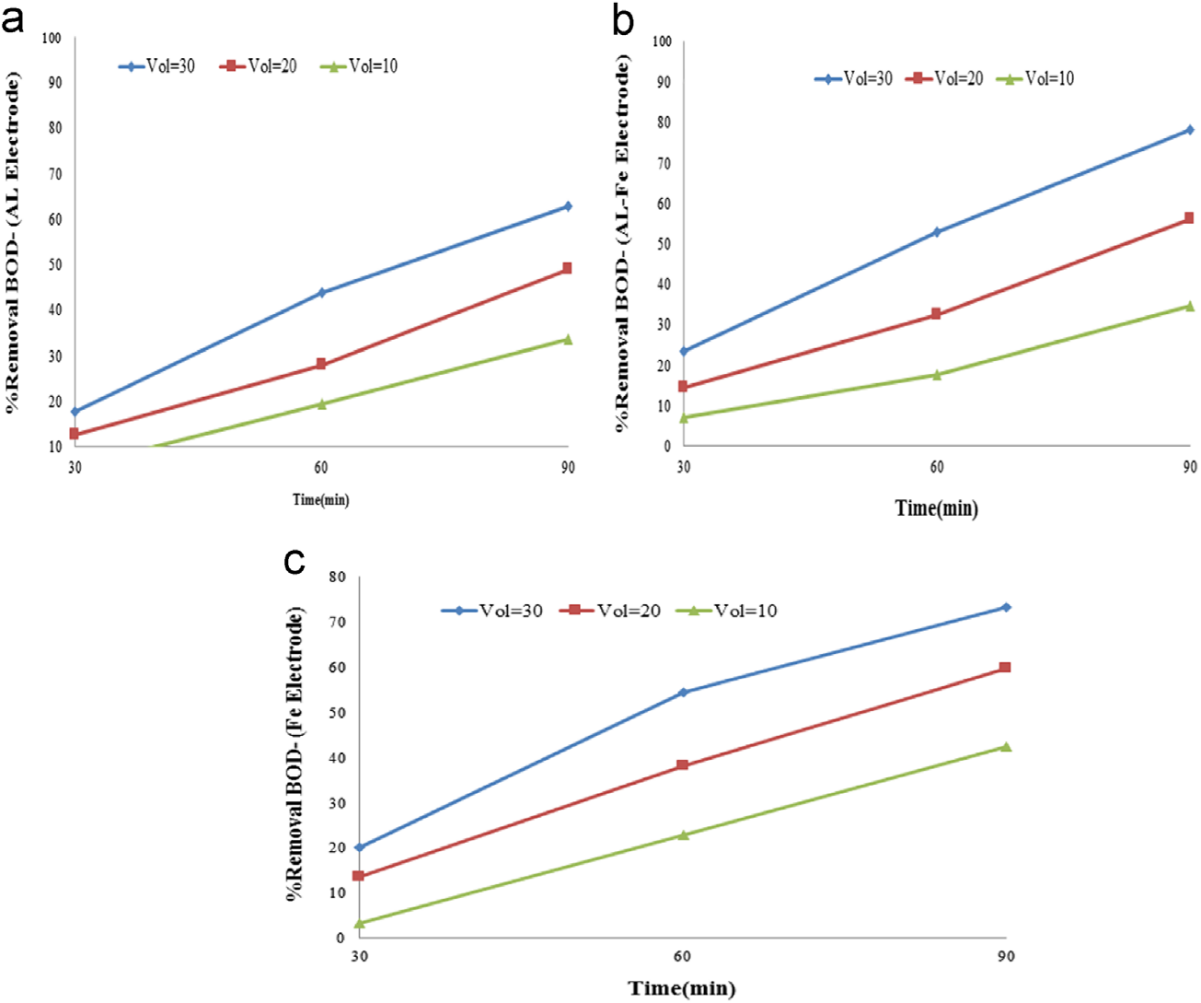
(a) Aluminum electrode, (b) Aluminum – Iron electrode, and (c) Iron electrode applied in the different Voltage on BOD removal efficiency .

**Fig. 3:**
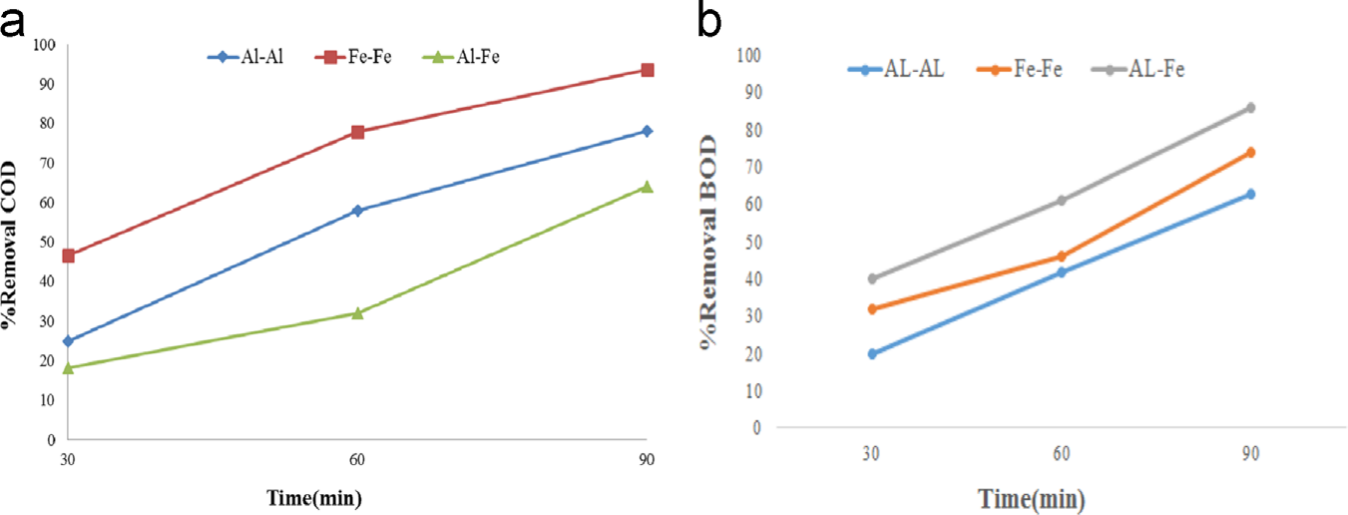
(a) Aluminum electrode, (b) Aluminum – Iron electrode, and (c) Iron electrode applied in the optimum pH=7 and voltage=30 on COD and BOD removal efficiency.

In this article the data in Table 1 present the measured parameters and characteristics of the raw grey water that used for description of experiments. Calculated values of K (1/min) and kWh/m^3^ in the grey water effluent are reported in Table 2. Fig. 1, Fig. 2 show data of different arrangements under optimal conditions applied in this study. The maximum removal efficiency (90.18%) of COD and BOD was obtained at optimum pH=7, level of 30 voltage, and contact time of 90 min. The effects of optimum parameters on removal efficiency of COD and BOD are shown in Fig. 3.

## 2 Experimental design, materials and methods

### 2.1 Sample collection and analytical procedures

Our data set was obtained from All Car washes. The raw grey water was obtained along the Ahvaz in Iran. The initial concentration of samples has been tested for determination of COD and BOD. To adjust the primary pH of the solution, the sulfuric acid and one-tenth normal sodium hydroxide were used. A lab-scale reactor with diameters of 15 cm×15 cm×15 cm was used for performing experiments. Sulfuric acid (H_2_SO_4_), potassium dichromate (K_2_Cr_2_O_7_), mercury sulfate (HgSO_4_), silver sulfate (Ag_2_SO_4_), potassium hydrogen phthalate (C_8_H_5_KO_4_), 3-methyl-2-benzothiazoline hydrazine were used for preparing COD and BOD solutions in grey water. Steering time of 30, 60 and 90 min, voltage values of 10, 20 and 30 v were used in this study. At each experiment, removal efficiency of COD and BOD in grey water with special Al–Al, Al–Fe, Fe–Fe electrode was investigated. Spectrophotometer (DR/5000 UV–vis HACH) was used to investigate the remaining concentration level of COD and BOD in the grey water effluent [5]. Following equation was applied to calculate the electrocoagulation electrical energy consumption during experiments [4], [5].

$$\frac{EE}{V}=\frac{U\times I\times t}{V_{r}}$$

where: *U* is voltage used in the process (V), *I* is intensity of the applied current (A), *t* is reaction time (min) and *V_r_* is reactor volume (Lit).

## Figures and Tables

**Table 1:** Parameters measured and characteristics of the raw carwash wastewater used for this study.

**Parameter**	**Range**	**Unit**	**Raw wastewater**
			**Mean±S.D**
**pH**	3, 7, 11	–	7.08±0.03
**Steering time**	30, 60, 90	min	–
**Voltage**	10, 20, 30	Volt	–
**Electrode type**	Al–Al, Fe–Fe, Al–Fe	–	–
**BOD**	–	(mg/L)	(102−246)±207.3
**COD**	–	(mg/L)	(480−1560)±207.3

**Table 2:** Electrode type, voltage, pH, K (1/min) and kWh/m^3^ values in the removal of COD and BOD in the present study.

**Electrode type**		**Voltage**	**pH**	**K (1/min)**	***kWh/m^3^***
**Fe–Fe**		30	7	14.15	*787.5*
			3	11.61	*1575*
			11	8.73	*2362.5*
		20	7	10.29	450
			3	9.76	900
			11	9.24	1350
		10	7	8.53	189
			3	4.43	378
			11	3.62	567
**Al–Al**		30	7	18.24	675
			3	13.92	1350
			11	15.10	2025
		20	7	14.58	330
			3	10.80	660
			11	10.51	990
		10	7	12.23	159
			3	8.27	318
			11	8.91	477
**Al–Fe**		30	7	13.99	900
			3	13.89	1800
			11	11.42	2700
		20	7	11.79	540
			3	9.77	1080
			11	11.30	1620
		10	7	9.76	240
			3	8.17	480
			11	6.21	720

K (1/min) is the rate constant of removal (1/min) related to the removal of COD and BOD.
