# Supplementary material for: Electrocoagulation process to Chemical and Biological Oxygen Demand treatment from carwash grey water in Ahvaz megacity, Iran
Source: Data Brief. 2017 Mar 9;11:634–9. doi: 10.1016/j.dib.2017.03.006 (PMC5369263; doi:10.1016/j.dib.2017.03.006)
Supplement: Supplementary file 1 — Supplementary material [file mmc1.doc]

**Electrocoagulation process to formaldehyde (CH_2_O) treatment from carwash grey water in Ahvaz megacity, Iran**

**Conflicts of Interest**

Authors have no conflicts of interest.

**Acknowledgment**

The authors of this research would like to extend their warm thanks to the Ahvaz Jundishapur University of Medical Sciences for their financial support.

**Funding/Support**

This work was financially supported by Ahvaz Jundishapur University of Medical Sciences.
